# Inhibition of Ceramide *De Novo* Synthesis Ameliorates Diet Induced Skeletal Muscles Insulin Resistance

**DOI:** 10.1155/2015/154762

**Published:** 2015-08-25

**Authors:** Krzysztof Kurek, Agnieszka Mikłosz, Bartłomiej Łukaszuk, Adrian Chabowski, Jan Górski, Małgorzata Żendzian-Piotrowska

**Affiliations:** Department of Physiology, Medical University of Bialystok, 2C Mickiewicza Street, 15-222 Białystok, Poland

## Abstract

Nowadays wrong nutritional habits and lack of physical activity give a rich soil for the development of insulin resistance and obesity. Many researches indicate lipids, especially the one from the sphingolipids class, as the group of molecules heavily implicated in the progress of insulin resistance in skeletal muscle. Recently, scientists have focused their scrutiny on myriocin, a potent chemical compound that inhibits ceramide (i.e., central hub of sphingolipids signaling pathway) *de novo* synthesis. In the present research we evaluated the effects of myriocin application on type 2 diabetes mellitus in three different types of skeletal muscles: (1) slow-oxidative (red gastrocnemius), (2) oxidative-glycolytic (soleus), and (3) glycolytic (white gastrocnemius). For these reasons the animals were randomly divided into four groups: “control” (C), “myriocin” (M), “high fat diet” (HFD), “high fat diet” (HFD), and “high fat diet + myriocin” (HFD + M). Our *in vivo* study demonstrated that ceramide synthesis inhibition reduces intramuscular ceramide, its precursor sphinganine, and its derivatives sphingosine and sphingosine-1-phosphate concentrations. Moreover, FFA and TG contents were also decreased after myriocin treatment. Thus, myriocin presents potential therapeutic perspectives with respect to the treatment of insulin resistance and its serious consequences in obese patients.

## 1. Introduction

Lipids and glucose are extremely important energy sources for mammalian body. Nowadays high accessibility of energy-dense food together with wrong nutritional habits and lack of physical activity gives a rich soil for the development of insulin resistance and obesity. Skeletal muscles due to their overall proportion in the construction of human body (up to ~40%) are the major contributor of whole body glucose-fatty acid balance and are the main place where the insulin resistance develops [1].

The relationship between bioactive lipid species accumulation and development of skeletal muscles insulin resistance is well established since it was confirmed in human, animal, and cell culture models [2, 3]. Lipids are heavily implicated in the progress of insulin resistance in skeletal muscle. This seems to be linked to an imbalance between bioactive lipid supply and its oxidation, the latter being related to reduced mitochondrial oxidative capacity in insulin resistant state. The important factors responsible for the pathogenesis of insulin resistance are intracellular accumulations of diacylglycerols (DAG) and triacylglycerols (TG) moieties. The accretion of intramuscular DAG content could be a result of prolonged, intensified free fatty acids (FFAs) release from insulin resistant white adipose tissue [4]. Data published so far confirmed strong positive relationship between increased intramuscular triacylglycerol (TG) content and the onset and development of insulin resistance. Moreover, intramuscular TG content is acknowledged as a marker of skeletal muscle insulin resistance [5].

Sphingolipids are a class of biologically active molecules that affects numerous aspects of cells functioning, ranging from their differentiation through proliferation to inflammatory processes and apoptosis [6]. Ceramide is a central molecule of sphingolipids metabolism and it can be generated in one of the four cellular synthesis pathways [6]. The most important of those routs is called* de novo* synthesis, and the other three encompass* salvage pathway*, sphingomyelin (SM), or ceramide-1 phosphate conversion. In the first step of* de novo* synthesis serine palmitoyltransferase (SPT) catalyzes condensation of palmitoyl-CoA and the amino acid, serine. As a result of this process 3-ketosphinganine and subsequently sphinganine (SFA) are produced. SFA is then acylated to form dihydroceramide, which is converted into ceramide by the addition of trans 4,5-double bond. Importantly, evidence exists that increased rate of ceramide* de novo* synthesis significantly contributes to the development of metabolic disorders such as insulin resistance or diabetes mellitus. Ceramide may be also cleaved, to the form of sphingosine, through the actions of ceramidases [7]. Interestingly, it seems that also ceramide derivatives, namely, sphingosine (SFO) or sphingosine-1-phosphate (S1P), do influence cellular growth and functions, and therefore, they also may be involved in the initiation and progression of metabolic diseases [8, 9].

Although a precise mechanism of ceramide influence on insulin signaling pathway needs further elucidation its role in the development of skeletal muscle insulin resistance has been postulated. Ceramide directly activates protein phosphatase 2A (PP2A), an enzyme responsible for deactivation (dephosphorylation) of Akt/protein kinase B (Akt/PKB) [10]. Moreover, ceramide blocks the translocation of the Akt/PKB from cytosol to plasma membrane [9]. Consistently, inhibiting ceramide synthesis in insulin resistant L6 myotubes restored their PKB/Akt phosphorylation and partially reversed their condition [11]. Moreover, it seems that also* in vivo* ceramide level is associated with insulin sensitivity in human. In a study of Straczkowski et al. [12] impaired insulin sensitivity and increased ceramide content were observed in the offspring of type 2 diabetic parents and in obese subjects in comparison with lean patients [12]. This data strongly suggest potential role of ceramide muscle accumulation in the development of skeletal muscle insulin resistance.

Commercially sourced from certain fungi species, most frequently* Mycelia sterilia*, a pharmaceutical termed myriocin is an effective and highly specific SPT inhibitor [13]. Some of these fungi were traditionally used in Asian medicine in an effort to achieve eternal youth [14]. Previously published studies provided evidences that SPT inhibitor, myriocin, may find potential application in the treatment of selected cardiovascular diseases, such as atherosclerosis [15, 16]. There are also some reports suggesting that postmyriocin reduction in ceramide level improves glucose homeostatic control and enhances whole body insulin responsiveness in animals' models of obesity and both types (1 and 2) of diabetes [17, 18]. Moreover, in recently published study we have revealed that treatment with myriocin reduced body weight, ameliorated glucose homeostasis, and reversed hepatic steatosis in rat model of nonalcoholic fatty liver disease [19]. Furthermore, we have demonstrated that inhibition of ceramide* de novo* synthesis with myriocin ameliorated glucose homeostasis in streptozotocin-induced type 1 diabetes animal model [20]. Summarizing the above data, myriocin can be considered as a potential new therapeutic tool for the treatment of selected metabolic diseases in the future. However, the effects of myriocin application with respect to intramuscular lipids profile and sphingolipids metabolism need further clarification. In the present study we evaluated the effect of SPT inhibitor, myriocin, on glucose homeostasis and lipid metabolism in the rat model of diet induced skeletal muscle insulin resistance.

## 2. Material and Methods

### 2.1. Animal Model

Animal maintenance and treatment conditions were approved by the Local Ethical Committee for Animal Experiments at the Medical University of Białystok. The following experimental procedures were conducted on male Wistar rats (8 rats in each group) kept in the appropriate animal husbandry facility. The animals had been provided with stable temperature (21-22°C), humidity, circadian rhythm (12 h/12 h light-dark cycle) and unrestricted,* ad libitum*, access to commercially available rodent chow and water. The distribution of the rats amongst experimental groups was performed at random (based on computer generated sequence of numbers). The four groups, referred to by code names, were as follows:

1. “C,”
2. “M,”
3. “HFD,”
4. “HFD + M.”

Group “C” was composed of control animals fed* ad libitum*. The “M” group was an experimental group treated with myriocin (intraperitoneal injections, dosage: 0.3 (mg/kg of body weight)) for 7 days. The rats from the “HFD” group “were on a 5-week high-fat diet” (Research Diets, Inc.; #D12492) composed in 60% of fats, 20% of carbohydrates, and 20% of proteins. Also the animals from the “HFD + M” group received that diet for 5 weeks; however, it was accompanied by simultaneous myriocin injections (7 days, since the fourth week). On the beginning of the 6th week the rats underwent overnight fasting followed by anesthetic procedure (intraperitoneal pentobarbital application (80 mg/kg of body weight)), sacrifice, and tissue collection. The tissue samples (soleus, red gastrocnemius and white gastrocnemius) were excised, frozen in liquid nitrogen, and stored at −80°C until further analyses. Blood samples were taken from abdominal aorta.

### 2.2. Glucose Homeostasis, Plasma Insulin, and Plasma Free Fatty Acids Concentration

According to the experiment protocol fasting serum glucose and insulin concentrations were evaluated. Glucose amount (mg/dL) was quantified with commercially available glucometer (Accu-Check, Bayer, Germany), whereas for the purpose of the plasma insulin level (*μ*U/mL) assessment chemiluminescence method was applied (Abbot, USA). Based on those measurements the homeostatic model assessment (HOMA) was determined, that is:

(1) HOMA=insulin concentration∗glucose concentration405.

Plasma free fatty acids concentration was determined using the method described by Bligh and Dyer [21].

### 2.3. Content of Skeletal Muscle Sphingomyelin

A precooled aluminum mortar was used for the samples pulverization and the resulting powder was placed into a tube with a mixture of methanol and 0.01% butylated hydroxytoluene (Sigma-Aldrich, Saint Louis, MO). In the subsequent step lipids were extracted according to the method proposed by Bligh and Dyer [21]. Briefly, a TLC (thin-layer chromatography) was applied for sphingomyelin isolation. The gel bands, after their reference to the standards, were abraded from the plates and moved into tubes containing pentadecanoic acid (Sigma-Aldrich). After subsequent transmethylation the sphingomyelin fatty acids were analyzed using gas-liquid chromatography (Hewlett-Packard 5890 Series II system, equipped with a double flame-ionization detector and Agilent CP-Sil 88 capillary column). Total sphingomyelin content was calculated as the sum of individual fatty acid species and is expressed in nM/g of the tissue.

### 2.4. Content of Skeletal Muscle Ceramide

A portion of lipids extracts, contained in the chloroform phase, was transferred to another tube containing C17-sphingosine (Avanti Polar Lipids, UK) as an internal standard. The organic phase containing ceramide was then hydrolyzed (in the solution of 1 M KOH in 90% methanol) for 60 min at the temperature of 90°C. The liberated sphingosine was subsequently analyzed using HPLC (high performance liquid chromatography). N-palmitoyl-sphingosine (Avanti Polar Lipids, UK) was used as a standard for preparation of the calibration curve. The content of ceramide was adjusted for the concentration of free sphingosine measured in the same sample.

### 2.5. Content of Skeletal Muscle Sphingosine, Sphinganine, and Sphingosine-1-Phosphate

Sphingolipid (SFO, SFA, and S1P) was measured as previously described by Min et al. [22]. Briefly, the samples mixed with C17-sphingosine and C17-S1P (added as internal standards, Avanti Polar Lipids, USA) underwent homogenization and ultrasonication. In the next step, and before their HPLC analysis (ProStar, Varian, USA), the sphingoid bases were transformed to their o-phthalaldehyde derivatives.

### 2.6. Skeletal Muscle Sphingomyelinase (Neutral and Acidic) Activity

The aforementioned enzymes activities were assessed using radiolabeled [N-methyl-14C]-SM as described by Liu and Hannun [23] and the resulting product (14C-choline phosphate) was placed to scintillation tubes and counted on Packard TRI-CARB TR scintillation counter. The results were adjusted for protein concentration (assessed prior enzymatic analysis with essentially fatty acids free bovine serum albumin (Sigma-Aldrich, USA) used as a standard).

### 2.7. Skeletal Muscle Ceramidase (Alkaline, Neutral, and Acidic) Activity

The activities of the ceramidases were determined according to Nikolova-Karakashian and Merrill Jr. [24] using radiolabeled [N-palmitoyl-1-14C]-sphingosine (Moravek Biochemicals, USA) and the resulting products (ceramide and 1-14C-palmitate) were separated with the use of basic Dole solution (isopropanol/heptane/1 N NaOH, 40,10,1; v/v/v). The 1-14C-palmitate radioactivity was measured on the scintillation counter and the results were normalized for protein concentration.

### 2.8. Content of Skeletal Muscle FFA and TG

A precooled aluminum mortar was used for the samples pulverization and the resulting powder was placed into a tube with a mixture of methanol and 0.01% butylated hydroxytoluene (Sigma-Aldrich, Saint Louis, MO). In the subsequent step lipids were extracted according to the method proposed by Bligh and Dyer [21]. Briefly, a TLC (thin-layer chromatography) was applied for total free fatty acids and triacylglycerols fractions separation. The identification of individual fatty acids methyl esters was performed based on the GLC standards retention times. The aforementioned procedure was conducted using gas chromatograph (Hewlett-Packard 5890 Series II) equipped with a Varian CP-SIL capillary column (50 m × 0.25 mm internal diameter) and flame-ionization detector (FID) (Agilent Technologies, CA, USA). Total FFAs and TAGs content was calculated as the sum of individual fatty acid species and is expressed in nM/g of the tissue.

### 2.9. Statistical Analysis

The results are presented in the form of bar plots (mean ± standard deviation (SD)). For the detection of the differences between the studied groups (8 replicates per group) one-way ANOVA with a subsequent post hoc test (Tukey HSD) was applied. Values were considered to be statistically significant at *α* = 0.05.

## 3. Results

### 3.1. Effects of High-Fat Diet and Myriocin Treatment on Body Weight, Glucose Homeostasis, and Plasma Free Fatty Acids Level (Table 1)

Average daily food intake (in g.) was comparable in all groups. Rats fed with high-fat diet were characterized by significant increase in body weight compared with control group (*p* < 0.05). On the other hand, animals from both groups treated with myriocin were characterized by significant body weight reduction in comparison with control and HFD groups (*p* < 0.05 and *p* < 0.05, resp.).

Moreover, we found that feeding with high-fat diet affected glucose homeostasis. We noticed increased fasting glucose as well as insulin level in HFD group compared to control animals (*p* < 0.05 and *p* < 0.05, resp.). Despite the fact that myriocin treatment did not cause any changes in fasting glucose and insulin concentrations in comparison with control group, rats fed with high-fat diet and treated with myriocin were characterized by significant decrease in fasting glucose and insulin levels compared with HFD group (*p* < 0.05 and *p* < 0.05, resp.). Further we observed that high-fat diet led to insulin resistance expressed by elevated HOMA-IR index in HFD group (*p* < 0.05), which was reversed after myriocin administration (*p* < 0.05).

In comparison with control group rats fed with high-fat diet were characterized by increased plasma free fatty acids concentration (*p* < 0.05). Similarly, in both groups treated with myriocin, significant increase in plasma FFA content was observed (*p* < 0.05 and *p* < 0.05, resp.).

### 3.2. Effect of High-Fat Diet and Myriocin Treatment on Skeletal Muscles Sphingolipids Content

**Figure 1:**
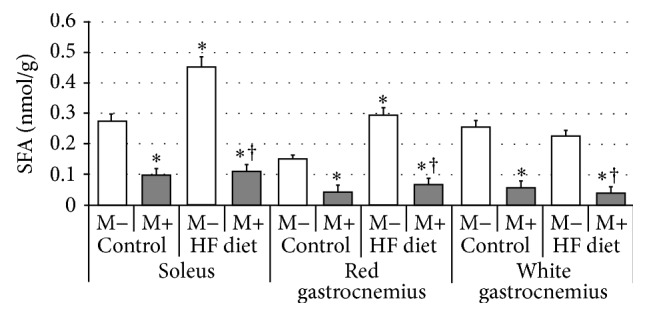
Effect of high-fat diet (HFD) feeding (5 weeks) and/or myriocin application (7 days) on sphinganine (SFA) content in skeletal muscles (*n* (per group) = 8). M+: rats administered with myriocin. M−: untreated group. Results are expressed as means ± SD. ^*∗*^ *p* < 0.05 compared with control group. ^†^ *p* < 0.05 compared with HF diet group.

**Figure 2:**
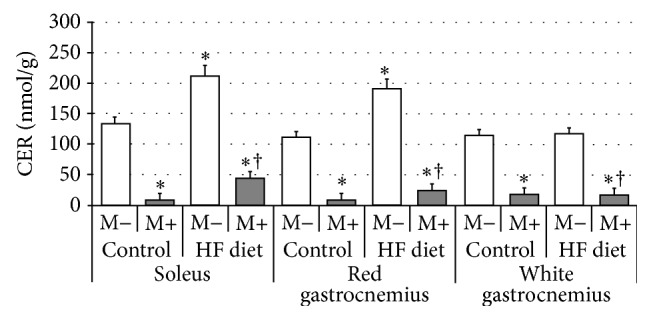
Effect of high-fat diet (HFD) feeding (5 weeks) and/or myriocin application (7 days) on ceramide (CER) content in skeletal muscles (*n* (per group) = 8). M+: rats administered with myriocin. M−: untreated group. Results are expressed as means ± SD. ^*∗*^ *p* < 0.05 compared with control group. ^†^ *p* < 0.05 compared with HF diet group.

**Figure 3:**
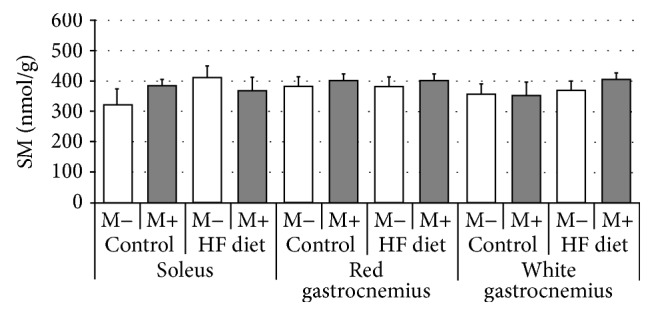
Effect of high-fat diet (HFD) feeding (5 weeks) and/or myriocin application (7 days) on sphingomyelin (SM) content in skeletal muscles (*n* (per group) = 8). M+: rats administered with myriocin. M−: untreated group. Results are expressed as means ± SD.

**Figure 4:**
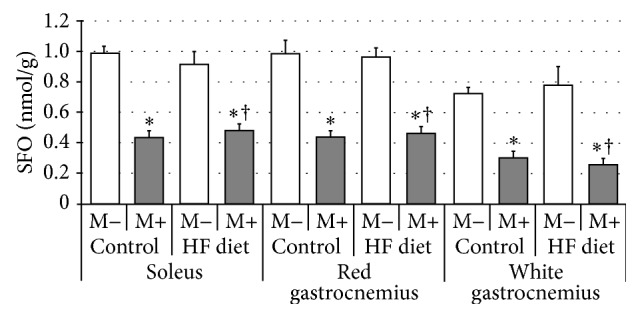
Effect of high-fat diet (HFD) feeding (5 weeks) and/or myriocin application (7 days) on sphingosine (SFO) content in skeletal muscles (*n* (per group) = 8). M+: rats administered with myriocin. M−: untreated group. Results are expressed as means ± SD. ^*∗*^ *p* < 0.05 compared with control group. ^†^ *p* < 0.05 compared with HF diet group.

**Figure 5:**
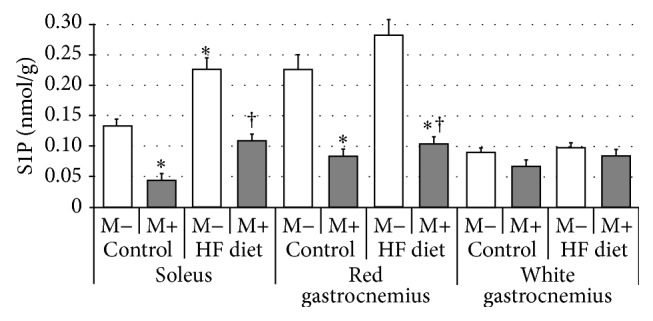
Effect of high-fat diet (HFD) feeding (5 weeks) and/or myriocin application (7 days) on sphingosine-1-phosphate (S1P) content in skeletal muscles (*n* (per group) = 8). M+: rats administered with myriocin. M−: untreated group. Results are expressed as means ± SD. ^*∗*^ *p* < 0.05 compared with control group. ^†^ *p* < 0.05 compared with HF diet group.

Compared with control group rats fed with high-fat diet were characterized by significant increase of SFA content in soleus and red gastrocnemius, but not in white gastrocnemius muscle (Figure 1, *p* < 0.05 and *p* < 0.05, resp.). After myriocin treatment a dramatic reduction in SFA level in comparison with control and HFD groups was noted in all three examined sections of skeletal muscle (Figure 1, *p* < 0.05). Subsequently, we found that high-fat diet feeding resulted in significant elevation of ceramide concentration in both soleus and red gastrocnemius, but not in white gastrocnemius muscle (Figure 2, *p* < 0.05 and *p* < 0.05, resp.). Treatment with myriocin significantly decreased ceramide content compared with control and HFD groups in soleus, red gastrocnemius, and white gastrocnemius muscle (Figure 2, *p* < 0.05). Surprisingly, we observed that sphingomyelin content remained unchanged after high-fat diet feeding as well as after myriocin treatment in all three sections of skeletal muscle (Figure 3). We found that high-fat diet had no influence on sphingosine content in any of the observed groups. However, groups receiving myriocin were characterized by significant reduction of SFO concentration in soleus, red gastrocnemius, and white gastrocnemius muscle (Figure 4, *p* < 0.05). Finally, we observed significant increment in sphingosine-1-phosphate content after high-fat diet feeding only in soleus muscle. Treatment with myriocin significantly decreased S1P concentrations in both soleus and red gastrocnemius muscles, but not in white gastrocnemius muscle (Figure 5, *p* < 0.05 and *p* < 0.05, resp.).

### 3.3. Effect of High-Fat Diet and Myriocin on the Activities of the Key Enzymes Regulating Sphingolipids Metabolism (Tables 2 and 3)

High-fat diet feeding has no significant effect on the activity of neutral sphingomyelinase. However, myriocin administration resulted in significant reduction of neutral sphingomyelinase compared with both control and rats fed with high-fat diet groups (Table 2, *p* < 0.05 and *p* < 0.05, resp.). Interestingly, we did not observe changes in acidic sphingomyelinase isoform activity in any type of skeletal muscles tested, neither in the “HFD” nor in the “HFD + M” group.

In comparison with control group high-fat diet feeding caused significant elevation of neutral and alkaline ceramidase activities in both soleus and red gastrocnemius muscles (Table 3, *p* < 0.05 and *p* < 0.05, resp.). Myriocin treatment resulted in decreased activity of neutral and alkaline ceramidase compared with control group and the group fed with high-fat diet in soleus and red gastrocnemius muscles (Table 3, *p* < 0.05 and *p* < 0.05, resp.). No significant changes (between any of the examined groups) in acidic ceramidase activity were observed in all types of rat skeletal muscles (Table 3).

### 3.4. Effect of High-Fat Diet and Myriocin Treatment on FFA and TG Content in Skeletal Muscles

**Figure 6:**
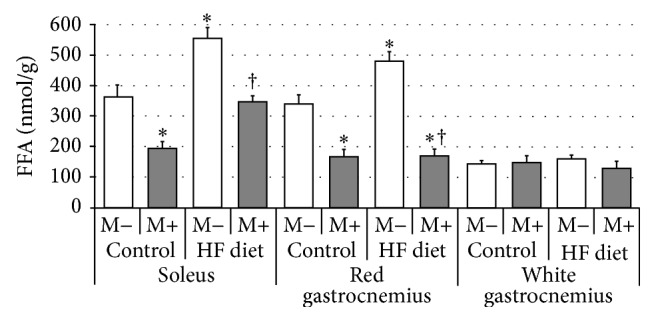
Effect of high-fat diet (HFD) feeding (5 weeks) and/or myriocin application (7 days) on free fatty acids (FFA) content in skeletal muscles (*n* (per group) = 8). M+: rats administered with myriocin. M−: untreated group. Results are expressed as means ± SD. ^*∗*^ *p* < 0.05 compared with control group. ^†^ *p* < 0.05 compared with HF diet group.

**Figure 7:**
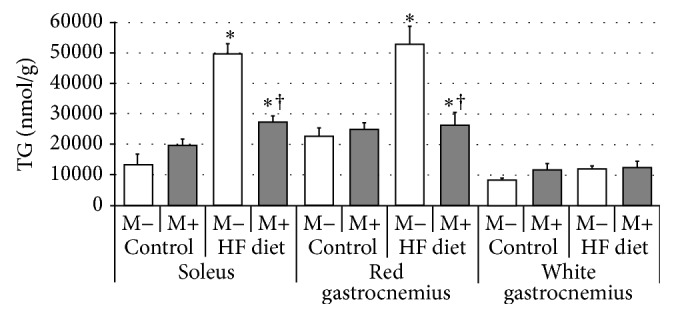
Effect of high-fat diet (HFD) feeding (5 weeks) and/or myriocin application (7 days) on triacylglycerols (TG) content in skeletal muscles (*n* (per group) = 8). M+: rats administered with myriocin. M−: untreated group. Results are expressed as means ± SD. ^*∗*^ *p* < 0.05 compared with control group. ^†^ *p* < 0.05 compared with HF diet group.

Compared with control group rats fed with high-fat diet were characterized by significant increase of FFA content in soleus and red gastrocnemius muscles, but not in white gastrocnemius muscle (Figure 6, *p* < 0.05 and *p* < 0.05, resp.). After myriocin administration a significant reduction in FFA level in comparison with HFD groups was observed in soleus and red gastrocnemius muscles (Figure 6, *p* < 0.05). Finally, we observed significant increment in TG content after high-fat diet feeding in soleus and red gastrocnemius muscles (Figure 7, *p* < 0.05 and *p* < 0.05, resp.). Treatment with myriocin significantly decreased TG concentration in both soleus and red gastrocnemius muscles (Figure 7, *p* < 0.05 and *p* < 0.05, resp.). No changes in TG level after high-fat diet feeding and myriocin administration (in white gastrocnemius muscle) were observed (Figure 7).

## 4. Discussion

Over nutrition and physical inactivity dependent obesity and type 2 diabetes frequently occur “hand in hand” and are commonly considered diseases of Western countries. Those conditions manifest themselves as hyperlipidemia, believed to be a major preceding event in the development of skeletal muscle insulin resistance [25, 26]. It has been proposed that skeletal muscle insulin resistance arises from the accumulation of intramuscular lipid metabolites (including free fatty acids, triacylglycerols, but mainly ceramide and its derivatives) that impede insulin signaling. Herein, we determined the role of ceramide* de novo* synthesis in mediating skeletal muscle insulin resistance.

Our results showed that the inhibition of ceramide synthesis in the muscle tissues improves the whole body glucose metabolism and is associated with a reversal of skeletal muscle insulin resistance induced by chronic high-fat feeding sustained for 5 weeks. After this period we have observed significant hyperglycemia and concomitant high fasting plasma insulin level. Furthermore, we have also noticed an increment in the “HFD” animals' body mas, whereas myriocin application had led to a significant weight loss despite similar food intake amongst all of the studied groups. In another study, performed on rodent model of diet induced insulin resistance, Yang et al. [27] suggested that body weight reduction after myriocin treatment depends on improved leptin signaling [27]. However, not all literature data confirm our findings. Ussher et al. [18] failed to observe the weight loss subsequent to myriocin administration. Perhaps this, somewhat interesting disagreement, was a result of different myriocin exposure times in both of those studies. Nevertheless, at least in our study, myriocin application did have other positive effects on the animals. Based on the HOMA-IR values we can claim that the inhibition of ceramide* de novo* synthesis substantially improved whole body insulin sensitivity. The aforementioned change was particularly evident in comparison of “HFD” and “HFD + M” groups and although 7 days were clearly not enough to achieve normal insulin level (similar to the control group) we believe that prolonged myriocin administration could totally reverse the observed insulin resistance. Moreover, this postmyriocin plasma hyperglycemia and insulin level normalizations are also consistent with the other literature data [17, 27].

In our study we noticed that intraperitoneal administration of SPT inhibitor, myriocin, caused several significant changes in sphingomyelin signaling pathway. First of all, we have observed that myriocin treatment significantly reduced ceramide* de novo* synthesis in rat skeletal muscles. This was manifested primarily by dramatic decrease in ceramide level and by a decrease in SFA (an important substrate for ceramide* de novo* synthesis) content in all types of skeletal muscles with both oxidative and glycolytic potentials: soleus, red gastrocnemius, and white gastrocnemius sections. Additionally, myriocin treatment inhibited activities of neutral and alkaline ceramidase, but not acidic ceramidase. This explains, therefore, observed after myriocin treatment reduction of SFO and S1P (ceramide downstream metabolites) content. In another study, Yang et al. [27] demonstrated modestly decreased SFO and S1P plasma concentration in myriocin treated rodents [27]. Surprisingly, we did not notice any significant changes in skeletal muscles sphingomyelin content, neither after chronic high-fat diet feeding nor after myriocin treatment. Moreover, myriocin exerted only slight effect on neutral isoform of sphingomyelinase and seemed to have virtually no influence on acidic sphingomyelinase. This strongly indicates that ceramide* de novo* synthesis pathway is a key mechanism in the regulation of overall ceramide metabolism. Moreover, it also points out its fundamental role in the development of metabolic syndrome and type 2 diabetes independently of the muscle fiber type [28].

Another important source for the synthesis of intracellular ceramide is free fatty acids. Increased supply of free fatty acids, especially palmitate, serves as a tool to induce enhanced ceramide synthesis, which in turn leads to impaired insulin responsiveness in oxidative (soleus and red gastrocnemius) skeletal muscles, liver, and/or adipose tissue [9, 19]. Thus, prevention against excessive circulating FFAs level may have potentially beneficial results with respect to insulin sensitivity. However, in our study we observed elevated FFAs plasma concentration after myriocin treatment with apparently no influence on whole body insulin resistance. Therefore, we postulate that being unable to utilize FFA (e.g.,* via* ceramide* de novo* synthesis pathway) free fatty acids may have undergone other routes, for example, as those responsible for DAG synthesis. These observations are consistent with previously published studies performed on insulin resistant L6 skeletal muscle cells [29]. The aforementioned authors revealed that in the absence of ceramide synthesis (reduced SPT activity) an increase in intramyocellular DAG and TG contents becomes a likely important determinant of insulin sensitivity [29].

Furthermore, another interesting and novel finding of our study is that administration of myriocin caused a reduction of intramuscular FFA and TG levels. Previously published studies have proven that intramuscular TAG accumulation has been associated with insulin resistance, although, this is now recognized as a marker of elevated intramuscular lipids rather than a direct cause of insulin resistance [30]. As expected, animals fed with high-fat diet were characterized by significant elevation of FFA and TG skeletal muscle content. Interestingly, we observed that inhibition of ceramide* de novo* synthesis resulted in decreased FFA and TG level in oxidative (soleus and red gastrocnemius) but not in glycolytic (white gastrocnemius) skeletal muscles. Moreover, this reduction was also associated with amelioration of whole body glucose homeostasis. These are advantageous findings, since previously published studies have postulated that skeletal muscle insulin resistance is caused by the intramyocellular accumulation of free fatty acids and TG [28]. Moreover, presented in this research observations are consistent with the results reported by other authors examining animal models of skeletal muscle insulin resistance [18]. However, it is important to emphasize that, in the opinion of many researchers, TG actually serve more as a buffer, protecting muscle cells against the accumulation of the more reactive lipid species [31, 32].

In conclusion, our* in vivo* study demonstrated, presumably for the first time, that administration of ceramide* de novo* synthesis inhibitor, myriocin, reduces intramuscular ceramide, its precursor sphinganine, and its derivatives sphingosine and sphingosine-1-phosphate concentrations. Moreover, FFA and TG contents were also decreased after myriocin treatment. This further resulted in body weight loss, amelioration of insulin resistance, and improvement of glucose homeostasis. It is tempting to speculate that limiting of intramuscular ceramide production and accumulation would confer an insulin-sensitizing effect. Thus, SPT inhibitor, myriocin, can find potential future application(s) as a therapeutic tool aimed at reducing insulin resistance and its serious consequences in obese patients.

## Figures and Tables

**Table 1:** Effect of myriocin treatment (for 7 days) and high fat diet feeding (for 5 weeks) on body weight, chow diet consumption, fasting serum glucose level, fasting serum insulin level, the Homeostasis Model Assessment of Insulin Resistance (HOMA-IR) index, and serum FFA level [*n* (per group) = 8].

	C	M	HFD	HFD + M
Initial body weight (g)	237.5 ± 10.7	209.9 ± 9.2	59.625 ± 5.37^*∗*^	59.75 ± 5.06^*∗*^
Final body weight (g)	315.6 ± 17.0	238.0 ± 17.4^*∗*^	375.4 ± 18.1^*∗*^	278.5 ± 14.3^*∗*†^
Chow consumption (g)	20.9 ± 3.6	19.3 ± 2.77	13.25 ± 1.39^*∗*^	13.63 ± 1.19^*∗*^
Glucose level (mg/dL)	101.3 ± 6.4	94.8 ± 7.5	164.5 ± 12.4^*∗*^	91.3 ± 11.6^†^
Insulin level (*μ*U/mL)	4.6 ± 0.6	4.5 ± 0.6	55.7 ± 5.7^*∗*^	34.8 ± 2.6^*∗*†^
HOMA-IR	1.6 ± 1.1	1.4 ± 0.6	20.0 ± 2.5^*∗*^	6.6 ± 1.3^*∗*†^
FFA level (*μ*mol/L)	88.6 ± 10.4	195.9 ± 22.6^*∗*^	152.4 ± 10.1^*∗*^	365.5 ± 17.6^*∗*†^

C: control group. M: group treated with myriocin. HFD: group fed with high fat diet. HFD + M: group fed with high fat diet and treated with myriocin. FFA: free fatty acids. Results are expressed as means ± SD.

^*∗*^ *p* < 0.05 compared with C group.

^†^ *p* < 0.05 compared with HFD group.

**Table 2:** Effect of myriocin treatment (for 7 days) and high fat diet feeding (for 5 weeks) on the neutral (nSMase) and acidic (aSMase) sphingomyelinases activities in rat skeletal muscle [*n* (per group) = 8].

	nSMase (nmol/h/mg protein)				aSMase (nmol/h/mg protein)			
	Control		HFD		Control		HFD	
	M−	M+	M−	M+	M−	M+	M−	M+
Soleus	4.6 ± 1.6	3.9 ± 0.8	4.7 ± 1.5	2.9 ± 0.9^*∗*†^	13.4 ± 3.4	14.6 ± 2.7	12.3 ± 2.1	14.1 ± 2.6
Red gastrocnemius	4.6 ± 1.2	5.2 ± 0.7	4.4 ± 0.8	2.6 ± 0.5^*∗*†^	6.7 ± 0.8	7.5 ± 1.4	7.2 ± 1.0	7.4 ± 1.3
White gastrocnemius	4.8 ± 0.6	5.7 ± 1.4	4.2 ± 1.0	3.2 ± 1.2^*∗*†^	4.5 ± 1.3	6.0 ± 1.6	5.4 ± 0.7	5.0 ± 1.6

HFD: group fed with high fat diet. M+: subgroup additionally treated with myriocin. M−: subgroup untreated with myriocin. Results are expressed as means ± SD.

^*∗*^ *p* < 0.05 compared with control group.

^†^ *p* < 0.05 compared with HFD group.

**Table 3:** Effect of myriocin treatment (for 7 days) and high fat diet feeding (for 5 weeks) on the neutral (nCer-ase), alkaline (alkCer-ase), and acidic (aCer-ase) ceramidases activities in rat skeletal muscle [*n* (per group) = 8].

	nCer-ase (nmol/h/mg protein)				alkCer-ase (nmol/h/mg protein)				aCer-ase (nmol/h/mg protein)			
	Control		HFD		Control		HFD		Control		HFD	
	M−	M+	M−	M+	M−	M+	M−	M+	M−	M+	M−	M+
Soleus	0.43 ± 0.2	0.16 ± 0.1^*∗*^	0.75 ± 0.2^*∗*^	0.15 ± 0.1^*∗*†^	0.55 ± 0.2	0.21 ± 0.1^*∗*^	0.87 ± 0.3^*∗*^	0.28 ± 0.1^*∗*†^	0.58 ± 0.3	0.45 ± 0.2	0.49 ± 0.2	0.51 ± 0.3
Red gastrocnemius	0.38 ± 0.2	0.24 ± 0.1^*∗*^	0.66 ± 0.1^*∗*^	0.21 ± 0.1^*∗*†^	0.48 ± 0.1	0.16 ± 0.1^*∗*^	0.78 ± 0.2^*∗*^	0.15 ± 0.1^*∗*†^	0.41 ± 0.2	0.48 ± 0.3	0.45 ± 0.1	0.52 ± 0.2
White gastrocnemius	0.17 ± 0.1	0.19 ± 0.1	0.16 ± 0.1	0.20 ± 0.1	0.24 ± 0.1	0.28 ± 0.1	0.18 ± 0.1	0.21 ± 0.1	0.21 ± 0.1	0.17 ± 0.1	0.20 ± 0.1	0.16 ± 0.1

HFD: group fed with high fat diet. M+: subgroup additionally treated with myriocin. M−: subgroup untreated with myriocin. Results are expressed as means ± SD.

^*∗*^ *p* < 0.05 compared with control group.

^†^ *p* < 0.05 compared with HFD group.
